# Phylogenomics of palearctic *Formica* species suggests a single origin of temporary parasitism and gives insights to the evolutionary pathway toward slave-making behaviour

**DOI:** 10.1186/s12862-018-1159-4

**Published:** 2018-03-28

**Authors:** Jonathan Romiguier, Jonathan Rolland, Claire Morandin, Laurent Keller

**Affiliations:** 1 Department of Ecology and Evolution, Biophore, University of Lausanne, 1015 Lausanne, Switzerland; 2 CNRS UMR-5554, Institut des Sciences de l’Evolution de Montpellier, Université de Montpellier, 34095 Montpellier, France; 3 Department of Zoology, University of British Columbia, #4200-6270 University Blvd, Vancouver, B.C. Canada; 4 Swiss Institute of Bioinformatics, Quartier Sorge, 1015 Lausanne, Switzerland; 5 Centre of Excellence in Biological Interactions, Department of Biosciences, University of Helsinki, Helsinki, Finland

**Keywords:** Ants, Formica, Phylogenomics, Social parasitism, Slave-making, Transcriptomes

## Abstract

**Background:**

The ants of the *Formica* genus are classical model species in evolutionary biology. In particular, Darwin used Formica as model species to better understand the evolution of slave-making, a parasitic behaviour where workers of another species are stolen to exploit their workforce. In his book “On the Origin of Species” (1859), Darwin first hypothesized that slave-making behaviour in *Formica* evolved in incremental steps from a free-living ancestor.

**Methods:**

The absence of a well-resolved phylogenetic tree of the genus prevent an assessment of whether relationships among *Formica* subgenera are compatible with this scenario. In this study, we resolve the relationships among the 4 palearctic Formica subgenera (*Formica str. s.*, *Coptoformica*, *Raptiformica* and *Serviformica*) using a phylogenomic dataset of 945 genes for 16 species.

**Results:**

We provide a reference tree resolving the relationships among the main *Formica* subgenera with high bootstrap supports.

**Discussion:**

The branching order of our tree suggests that the free-living lifestyle is ancestral in the *Formica* genus and that parasitic colony founding could have evolved a single time, probably acting as a pre-adaptation to slave-making behaviour.

**Conclusion:**

This phylogenetic tree provides a solid backbone for future evolutionary studies in the *Formica* genus and slave-making behaviour.

**Electronic supplementary material:**

The online version of this article (10.1186/s12862-018-1159-4) contains supplementary material, which is available to authorized users.

## Background

From birds to insects, many organisms can reduce the costs of brood rearing by exploiting resources from other species [1]. Certain ant species display an advanced form of parasitim, social parasitism, whereby two species of social insects coexist in the same nest, one of which is parasitically dependent on the other [2]. Slave-making in ants is a spectacular case of social parasitism. For example, the slave-making ant *Formica sanguinea* infiltrates nests of its slaves (e.g. the ant *Formica fusca*) to capture brood that are then reared inside the nest of the slave-making ant. After eclosion, the slave will perform typical worker tasks such as foraging and defending the colony [3]. In ants, slave-making behaviour is believed to have evolved nine times within two of the 21 known subfamilies, the *Formicinae* and the *Myrmicinae* [2, 4, 5]. In fact, only 0.5% of the known ant species are active slave makers [6], and the origins of slave-making in ants are still not well understood. The *Formica* genus is historically renowned as a classical model for studying the evolution of social parasitism [6–8]. Reflecting the importance of social parasitism in the genus, the classic taxonomic division of *Formica* in four subgenera is partly based on host/parasite status.

Palearctic *Formica* species are classically divided in four subgenera. The first subgenus, *Serviformica* (derived from the latin servire: “be a servant, be enslaved”), comprises many free-living species that are used as hosts by the three other subgenera. Contrary to the other subgenera, a single *Serviformica* queen can found a new colony independently. The second and third subgenera, *Coptoformica* and *Formica s. str*, are often referred to as “wood ants” and have a similar ecology. They build large mounds from plant material and can start new colonies by budding or temporary parasitism. Budding is a process whereby new queens and workers leave the mother to initiate a new colony nearby. This strategy is particularly common in species forming supercolonies consisting of many inter-connected nests [9–11]. In the case of temporary parasitism, newly-mated queens enter the nest of a *Serviformica* host species where they expel and replace the original queen to use the host workers as helpers. Host workers are then gradually replaced by the daughters of the temporary parasite queen. Finally, the fourth subgenus (*Raptiformica*, derived from the latin raptus: “to seize”) contains the only *Formica* species that practice slave-making, which is the most spectacular form of social parasitism in the genus. During a process called slave-raiding, *Raptiformica* workers capture brood of *Serviformica* species to increase the worker force of their own colony. After emerging in the slave-maker nest, the *Serviformica* workers behave as if they were in their own colony. Seasonal slave-raiding allows a continuous replenishing of slaves from neighboring host nests. In addition to slave-raiding, all species of the subgenus *Raptiformica* also initiate new colonies by temporary parasitism, similarly to *Coptoformica* and *Formica s. str.* Only one species of *Raptiformica* lives in the palearctic region (*F. sanguinea*, which is the type-species of the subgenus), while all other species (11) are found in the nearctic region [12].

The evolutionary pathway toward slavery has been extensively discussed [5, 8, 13–17]. In his book “*On the Origin of Species”* [8], Darwin first suggested that slave-raiding in the genus *Formica* might evolve progressively through an intermediary step of brood predation whereby some individuals would not be eaten and thus lead to accidental “slave-making”. Building on the idea of gradual evolution from free-living lifestyle to slave-making, Santschi [18] suggested that temporary parasitic colony founding is an intermediary step towards slave-making. During parasitic colony founding, the queen uses workers of other species as helpers, which may facilitate the use of slaves acquired after raids. By contrast, Wheeler [17] proposed that parasitic colony founding evolved several times independently in *Formica* and is not an intermediary step toward slavery*.* Finally, Buschinger [14] proposed that brood transport among nests of a multi-nest colony (i.e., polydomy) acted as an early step towards brood robbing, as seen in slave-raiding. Alloway [16] extended this theory by suggesting that brood exchange among nests of a multi-nest colony evolved toward selfish brood robbing during territorial battles. Such intra-specific brood robbing could have ultimately led to the inter-specific slave-raiding observed in *Raptiformica* species*.*

Discriminating between these hypotheses requires a robust phylogeny of the genus *Formica*. Several molecular phylogenetic studies have tried to resolve the phylogeny of palearctic *Formica* [19–21], but the relationship among subgenera is still unclear, probably because of the low number of loci used for these studies (e.g. allozymes and the *cytb* mitochondrial gene). A resolved phylogenetic tree of the subgenera is necessary to answer two key questions regarding the evolutionary pathway toward slavery in the *Formica* genus. The first is whether the ancestral lifestyle of the *Formica* genus is similar to the free-living *Serviformica* species. This question could not be answered till now, because the exact position of *Serviformica* in the tree was unknown and the monophyly of this subgenus has also never been clearly supported by molecular data [20]. The second question is whether parasitic colony founding did evolve once or repeatedly. A monophyletic clade grouping all social parasites (subgenera *Raptiformica*, *Coptoformica* and *Formica s. str*) would suggest a single origin of temporary parasitism in *Formica*, supporting the idea that parasitic colony founding has been a prerequisite for slave-making to emerge in *Raptiformica* [18]. Alternatively, if these three subgenera of social parasites do not form a monophyletic clade, this would instead support the view that temporary parasitism and slave-making are not evolutionarily tied and evolved several times independently [17]. Because *Raptiformica* slave-makers and wood ants *Coptoformica* and *Formica s. str.* Often build multi-nest colonies (*i.e* polydomy) [9], a clade grouping these three subgenera would also provide support to the theory that brood raiding of *Raptiformica* slave-makers is derived from brood transport among nests of a polydomous colony, as suggested by several authors [5, 14, 16].

To reconstruct a robust phylogeny of the *Formica* genus, we generated a large transcriptomic dataset including 10 different species from the four *Formica* subgenera (*Formica s. str.*, *Coptoformica, Raptiformica* and *Serviformica*). We completed our phylogenomic dataset with six *Formica* transcriptomes available from the literature [22], and resolved the deepest nodes of the *Formica* tree, giving insight to the evolutionary pathway toward slavery in the *Formica* genus.

## Methods

### Sampling and RNA extraction

We sampled a total of 10 species (*F. gagates*, *F. fusca*, *F. selysi*, *F. rufibarbis*, *F. cunicularia*, *F. sanguinea*, *F. pratensis*, *F. paralugubris*, *F. polyctena* and *F. bruni*) distributed among the 4 palearctic subgenera *Formica s. str.*, *Coptoformica, Raptiformica* and *Serviformica* and used one *Polyergus* species (*P. rufescens*) as an outgroup to root our phylogeny. The whole body of one individual of each species was flash-frozen in liquid nitrogen then stored at − 80 °C before RNA-extraction. Total RNA was extracted using specific protocols for ants [23]. Main RNA-extraction steps of this protocol were tissue disruption, lysate homogenization, isolation and purification of RNA. Prior to precipitation of the RNA with isopropanol, 10 μg of RNAase-free glycogen was added to the aqueous phase to increase the RNA yield. We used a NanoDrop spectrophotometer and an Agilent 2100 Bioanalyzer to check the quantity and the integrity of RNA extractions.

### Transcriptome sequencing and assembly

Complementary libraries were prepared using Illumina TrueSeq preparation kit. These libraries were sequenced on a HiSeq 2000 (Illumina) to produce 100-base-pairs (bp) paired-end reads. We used Trimmomatic to remove adapters and reads with length less than 60 bp and average quality less than 30 [24]. De novo transcriptome assemblies were performed using a combination of *ABySS (Assembly By Short Sequences)* and *Cap3*, following the strategy of Romiguier et al. [25]. The contigs generated by *ABySS* were used in two consecutive *Cap3* runs. Illumina reads of all individuals were mapped to the de novo transcriptome assembly of its corresponding species using the *BWA* program [26]. The contigs with a per-individual average coverage below X2.5 were discarded.

### Ortholog genes and alignments

We used the *Trinity* package [27] to predict Open Reading Frames (ORFs) and discarded ORFs shorter than 200 bp. In contigs with ORFs longer than 200 bp, 5′ and 3′ flanking non-coding sequences were deleted, thus producing predicted coding sequences that are hereafter referred to as genes. We performed this coding sequence detection on our 11 (10 *Formica* + 1 *Polyergus*) species and repeated the same procedure on 5 supplementary species (namely *F. exsecta, F. pressilabris, F. truncatulus, F. aquilonia* and *F. cinerea*) with transcriptomes available from a recent article [22]. We used *OrthoMCL* [28] to retrieve 945 one-to-one ortholog genes among these 16 species. We then aligned all these ortholog genes using *MACSE*, a multiple sequence alignment software that aligns nucleotide sequences with respect to their amino-acid translation [29]. We set the options with a cost of 10 for frameshift and 60 for stop codons, as advised by the user manual for transcriptomic data [29].

### Phylogenetic analyses

We performed phylogenetic analyses using three different methods: Maximum likelihood methods (*RAxML*) [30], Bayesian methods (*PhyloBayes*) [31] and coalescence methods (*MP-EST*) [32]. Maximum likelihood and Bayesian inferences are the two most common probabilistic tree reconstruction methods, and were used on large alignments of concatenated genes (supermatrix approach). Coalescence methods have a different but complementary philosophy and infer a species tree from multiple gene trees (supertree approach). All computations were performed at the Vital-IT (http://www.vital-it.ch) Center for high-performance computing of the SIB Swiss Institute of Bioinformatics.

#### Maximum likelihood (RAxML)

We concatenated all the ortholog genes in a single supermatrix alignment of 1270,080 bp (referred later as the *ALLPOSITIONS* supermatrix), then refined this supermatrix using the automated method implemented in *trimal* [33] to obtain a supermatrix of 970,619 bp (referred later as the *CLEAN* supermatrix). We also used a stricter cleaning procedure by eliminating all nucleotide positions containing a gap in at least one of the 16 species, reducing the size of the alignments to 621,307 bp (referred later as the *GAPLESS* supermatrix). As genes with high GC-content may dramatically bias tree reconstruction [34, 35], we also used an alignment concatenating only the 50% most GC-poor genes of the dataset (472 genes, total of 647,706 bp, referred later as the *GCPOOR* supermatrix). The *CLEAN*, *GAPLESS* and *GCPOOR* supermatrices were analyzed with *RAxML* [30] using a GTR + GAMMA model with 500 bootstrap replications. We compute a supplementary tree by partitioning the *ALLPOSITIONS* supermatrix by codon positions (i.e. different parameter estimation for the sites belonging to the 1st, 2nd or 3rd codon position) using RAxML and a GTR + GAMMA model (500 bootstrap replications).

#### Bayesian method (PhyloBayes)

For Bayesian inference we used *PhyloBayes MPI* [31] with a CAT-GTR model. This model takes into account site-specific nucleotide preferences, which better models the level of heterogeneity seen in real data and is well suited to large multigene alignments [36, 37]. Because this method is computationally more costly than a maximum likelihood approach (RAxML), it was only run using the GAPLESS supermatrix (621,307 bp). We run two independent Markov chains and convergence was assessed by comparing the two independent Markov chains with *bpcomp* and *tracecomp* tools from *PhyloBayes*. We stopped the inferences after 15,000 generations, with a maximum discrepancy in clade support of 0 (*maxdiff* metrics from *bpcomp*), a minimal effective sample size of 50 (*effsize* metrics from *tracecomp*) and a maximal relative difference in posterior mean estimates of 0.3 (*red_diff* metrics from *tracecomp*). The appropriate number of generations to discard as “burn-in” (1000) was assessed visually using *Tracer 1.6*.

#### Coalescence based method (*MP-EST*)

Recently developed coalescence-based methods use multiple gene trees to reconstruct phylogenies. Contrary to the other phylogenetic methods used in this article, this method does not use a concatenated sequence of all the genes but builds a species tree based on every individual gene tree. The main advantage of this approach is to better take into account incomplete lineage sorting [32, 38], a phenomenon whereby different gene trees differ from the species tree [39]. We used *MP-EST* (Maximum Pseudolikelihood Estimation of the Species Tree), a coalescence-based method that estimates a species tree from a set of gene trees by maximizing a pseudo likelihood function [32]. We built individual gene trees with *RAxML* (GTR + GAMMA model, 500 bootstrap replicates) and used the resulting 500 bootstrap replicates of each gene tree (available as supplementary material) to compute a species tree with *MP-EST* through the *STRAW* web server [40].

#### SH tests of monophyly

To test for the monophyly of the *Serviformica* subgenera, we performed Shimodaira-Hasegawa tests [41] as implemented in *RAxML*. We used the *CLEAN* supermatrix to compare the maximum likelihood value of a tree that constrains the monophyly of *Serviformica* species to the maximum likelihood value of the best unconstrained tree.

## Results

### Phylogenetic analyses

**Fig. 1:**
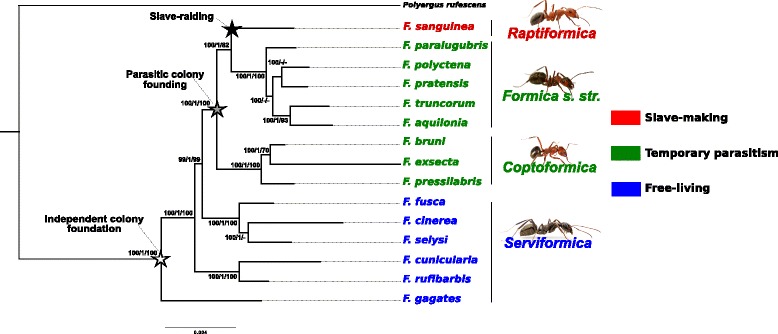
Molecular phylogeny of *Formica*. Branch lengths and topology are based on the Maximum-likelihood analysis (*CLEAN* supermatrix). Support of the three methods (Maximum-likelihood, Bayesian and Coalescence-based method) is indicated for each node. When a method does not retrieve a node, the support value is replaced by a “-”

We generated a phylogenomic dataset of 965 ortholog genes in 16 species that we concatenated in a single multi-gene alignment cleaned using three different procedures (*CLEAN*, *GAPLESS* and *GCPOOR*, see Material and Methods for details) and analysed these data (supermatrices or individual gene trees) with three different phylogenetic methods (maximum likelihood with *RAxML*, bayesian inference with *PhyloBayes* and a supertree coalescence-based method with *MP-EST*, see Material and Methods for details). All analyses retrieved essentially the same phylogenetic relationships with only few discrepancies. These discrepancies concerned relationships among highly related species, in particular in the *Formica str. s.* subgenus (Fig. 1). This result is not surprising given that there are many cases of hybrids in this taxonomic group and even colonies may comprise several species of this subgenus [42–48]. It is likely that hybridization is associated with significant gene flow among species, which, in turn, will cause discrepancies among gene trees and thus hamper species tree reconstructions, regardless of the method used [49]. Bayesian inference (*PhyloBayes*) recovered the highest support values while the coalescence-based approach (*MP-EST*) retrieved globally slightly lower support values (Fig. 1). Phylogenetic trees retrieved for each analysis are available in the Supplementary Material section (Additional file 1: Fig. S1, Additional file 2: Fig. S2, Additional file 3: Fig. S3, Additional file 4: Fig. S4, Additional file 5: Fig. S5 and Additional file 6: Fig. S6). The tree of the RAxML + *CLEAN* analysis is used as the reference for the topology and branch lengths in Fig. 1 while the nodal support of the bayesian (*PhyloBayes*) and coalescent-based approach (*MP-EST)* are mapped on each node. Exactly the same topology is obtained by partitioning the dataset by codon positions (RAxML + ALLPOSITIONS analysis, Additional file 6: Fig. S6).

### Non-monophyly of *Serviformica*

Our results do not support the monophyly of the subgenus *Serviformica.* Phylogenetic analyses of the six species of this subgenus indicate with high support values that these species are clustered in three different monophyletic clades (Fig. 1), namely (*F. fusca* + *F. cinerea + F. selysi*), (*F. cunicularia* + *F. rufescens*) and (*F. gagates*). To further validate the non-monophyly of the *Serviformica* subgenus, we performed a Shimodaira-Hasegawa test [41] by comparing the likelihood of a tree constraining the monophyly of the six *Serviformica* species with the likelihood of the unconstrained tree retrieved in the RAxML + *CLEAN* analysis. The likelihood of the unconstrained tree was significantly higher than the likelihood of the tree constraining the monophyly of *Serviformica* (respectively − 1,865,962 and − 1,867,685, SH test *p*-value < 0.01), confirming the non-monophyly of *Serviformica*.

### Monophyly of social parasites

All the analyses support with maximal values the monophyly of the *Coptoformica* and *Formica str. s.* subgenera (Fig. 1). This result confirms previous phylogenetic studies [19, 20]. More interestingly, we also retrieved a monophyletic clade grouping together the temporary social parasite subgenera *Coptoformica*, *Formica s. str* and *Raptiformica*. The support for this grouping is unambiguous and maximal in all the phylogenies constructed in our study (100 in the three *RAxML* Maximal likelihood analyses, 1.0 for the *PhyloBayes* Bayesian inference and 100 for the *MP-EST* shortcut coalescence approach). This result contrasts with previous studies that failed to retrieve a high bootstrap support for the monophyly of the temporary social parasite clade [19, 20]. *Coptoformica*, *Formica s. str.* and *Raptiformica* subgenera share important ecological traits, such as the loss of the ability to independently found new colonies and temporary parasitic colony founding. A single clade grouping these subgenera suggests that they inherited the ability to parasite *Serviformica* nests from a common ancestor. This result suggests a common origin of social parasitism in both wood ants (*Formica str. s.* and *Coptoformica*) and slave-makers (*Raptiformica*).

### Phylogenetic position of Nearctic *Formica* species

Although our species sampling includes all described palearctic *Formica* subgenera, it lacks representatives of nearctic species, particularly species of two described nearctic groups of slave species, namely the *F. neogagates* group and the *F. pallidefulva* group [12]. To confirm that these two nearctic groups of slave species do not belong to the clades of social parasites (*Formica str. s*, *Coptoformica* and *Raptiformica*), which may affect our conclusion of a single origin of slave-making, we built an additional phylogeny based on the *cox1* sequence of all Formica species available in GeneBank (i.e., 41 species, 19 with a nearctic distribution). As expected by the short length of the alignment (1270 bp), the resulting phylogenetic tree (Additional file 7: Fig. S7) has few well-resolved nodes (i.e. bootstrap support > 70), but there is good support for the *F. neogagates* group (represented by *F. neogagates*, *F. perpilosa* and *F. lasioides*) and the *F. pallidefulva* group (represented by *F. pallidefulva*) being not part of the parasitic clades. Rather, these two groups appear to be the two most basal clades of this *Formica* phylogeny (supported by a bootstrap of 87). Among the other well-resolved phylogenetic relationships, this analysis also retrieved three clades corresponding to the three social parasites subgenera, namely *Raptiformica* (bootstrap of 94), *Formica str. s.* species (bootstrap of 87) and *Coptoformica* species (bootstrap of 94). Importantly, all the nearctic *Raptiformica* species (*F. wheeleri, F. aserva* and *F. subintegra*) cluster with the palearctic *F. sanguinea.* This well-supported monophyly of the morphologically-defined *Raptiformica* subgenus thus indicates that slave-raiding did not evolve independently in the palearctic and nearctic regions, supporting the view of a single origin of slave-making in the *Formica* genus*.*

## Discussion

The six species of the subgenus *Serviformica* clustered in three different monophyletic clades (Fig. 1). Previous studies already questioned the monophyly of Serviformica, but the low number of molecular markers prevented sufficiently high support values (> 70) to give a clear answer [20, 21]. Our results, which are based on a large phylogenomic dataset, demonstrate that Serviformica should not be considered as a subgenus anymore, but is a paraphyletic group of species occupying a basal position in the Formica genus. Because all Serviformica species are free living (i.e., able to start new colonies on their own), this indicates that a free living lifestyle is a shared ancestral state (i.e. plesiomorphy) of Serviformica species, and then is the ancestral state of the Formica genus.

Our results are consistent with two previous theories proposed to explain the evolution towards slavery in *Formica*. The first is that parasitic colony founding is an intermediary step from independent colony founding to slave-making [15]. The second is that brood transport among nests of polydomous colonies preceded brood robbing observed in slave-raiding [14]. The branching order of our phylogeny suggests an evolutionary pathway toward slavery in several steps. The basal position of *Serviformica* species in the *Formica* phylogeny suggests a free-living ancestor with independent colony founding (white star in Fig. 1). From this ancestral state, our phylogenetic trees support a single loss of independent colony founding (grey star in Fig. 1) in both wood ants *(Coptoformica* and *Formica s. str.) a*nd slave-makers (*Raptiformica*). Dependent colony founding has been suggested as an adaptation to unfavorable cold habitat where success of independent colony founding is limited by high queen mortality [2, 6, 50]. This is supported by the alpine/boreal distribution of *Formica* social parasites and the fact that they all build mound nests from plant materials, which is known to increase thermal isolation [51]. To adapt to cold habitats, the ancestor of *Formica* social parasites may have avoided independent colony founding by allowing the return of mated queens in the parental colony, a hypothesis supported by the high occurrence of polygyny in the social parasite clades *Raptiformica*, *Coptoformica* and *Formica s. str*. As suggested by Buschinger [14], parasitic colony founding is then likely to have evolved from a state where queens returned to an established nest of their species to exploit the workforce and the security of other species nests. The finding that the *Raptiformica* slave-maker subgenus is nested in the monophyletic clade grouping the two wood-ant subgenera (*Formica s. str* and C*optoformica*) suggests that slave-raiding evolved at some point from a wood-ant ancestor (black star in Fig. 1). As typically seen in both wood-ant and *Raptiformica* species, such an ancestor of the *Raptiformica* slave-makers is likely to have featured polydomous (multi-nests) colonies, as suggested by Buschinger’s hypothesis [14] whereby slave-raiding evolved from opportunistic brood transport among nests of large polydomous colonies.

While our phylogenomic dataset offers an unprecedented amount of genetic information for the *Formica* genus (up to 1270,080 bp), one of its limitations is the exclusively palearctic distribution of the species sampled. This sampling issue is unlikely to affect our conclusions regarding the non-monophyly of *Serviformica*, but can affect our conclusions regarding the monophyly of social parasites (*Formica s. str.* + *Coptoformica + Raptiformica*). Based on our analysis of the *cox1* sequence of 41 species (including a total of 19 nearctic species, Additional file 7: Fig. S7), we can however reasonably exclude the possibility that nearctic groups of slave species (*F. pallidefulva* group and *F. neogagates* group) cluster with social parasites. Furthermore, most of the social parasite species (19 out of 22) are clustered in their expected social parasite subgenus, namely *Coptoformica, Formica s. str.* or *Raptiformica* (Additional file 7: Fig. S7). However, this gene analysis of a single gene does not allow one to give a clear position of *F. uralensis, F. dakotensis* and *F. ulkei,* three species that have been reported to practice temporary parasitism during colony founding [2]***.*** These species are traditionally thought to be part of the *Formica s. str*. Subgenus (for *F. uralensis* and *F. dakotensis*) or the *Coptoformica* subgenus (*F. ulkei*), but their subgenus affiliation is here not confirmed, an issue already known for *F. uralensis* that has a notoriously controversial phylogenetic position [19, 20]. Future phylogenomics dataset analyses should include these controversial species in order to clarify their position in the *Formica* phylogeny and confirm whether parasitic colony founding appeared only once in the genus.

## Conclusion

This study resolves the phylogenetic relationships among palearctic *Formica* subgenera. Interestingly, our phylogenetic tree reveals that the free-living *Serviformica* species do not form a monophyletic clade, and that parasitic colony founding in wood ants and *Raptiformica* slave-makers is likely to have a single origin. Slave-making behaviour is observed in nine different ant genera and has evolved several times repeatedly across the ant phylogeny [6]. While slave-maker species and slave species tend to be closely related [52], the evolutionary origins of slave making itself remains obscure. Our results suggest that parasitic colony founding is likely to be an intermediary step between free-living hosts and slave-maker parasites in the *Formica* genus. Similar studies in other genera containing slave-making species (e.g. *Temnothorax*, *Harpagoxenus*, *Myrmoxenus*, *Protomognathus…*) will be necessary to get a better global picture of the evolution of slave-making in ants.

## Additional files

Additional file 1 Figure S1 Phylogenetic tree of the *CLEAN* supermatrix (970,619 bp) built using *RAxML* (GTR + GAMMA model, 500 bootstrap replications). (PDF 2 kb)

Additional file 2 Figure S2 Phylogenetic tree of the *GAPLESS* supermatrix (621,307 bp) built using *RAxML* (GTR *+* GAMMA model, 500 bootstrap replications). (PDF 2 kb)

Additional file 3 Figure S3 Phylogenetic tree of the *GCPOOR* supermatrix (647,706 bp) built using *RAxML* (GTR + GAMMA model*,* 500 bootstrap replications). (PDF 2 kb)

Additional file 4 Figure S4 Phylogenetic tree of the *GAPLESS* supermatrix (621,307 bp) built using *PhyloBayes* (two independent Markov chains, 15,000 generations). (PDF 2 kb)

Additional file 5 Figure S5 Phylogenetic tree of the *MP-EST* analysis based on 945 gene trees (500 bootstrap replications for each gene tree). (PDF 2 kb)

Additional file 6 Figure S6 Phylogenetic tree of the *ALLPOSITIONS* supermatrix (1270,080 bp) built using *RAxML* by partitioning the supermatrix by codon positions (GTR + GAMMA model, 500 bootstrap replications). (PDF 2 kb)

Additional file 7 Figure S7 Phylogenetic tree based on the *cox1* mitochondrial gene of 41 *Formica* species borrowed from GeneBank (NCBI ID indicated between parentheses). The tree was built using *RAxML* (GTR + GAMMA, 500 bootstrap replications). Nodes supported by a bootstrap inferior to 70 were removed. Nearctic species are highlighted in red. (PDF 35 kb)
