# Supplementary figures and images for: Phylogenomics of palearctic Formica species suggests a single origin of temporary parasitism and gives insights to the evolutionary pathway toward slave-making behaviour
Source: BMC Evol Biol. 2018 Mar 28;18:40. doi: 10.1186/s12862-018-1159-4 (PMC5872393; doi:10.1186/s12862-018-1159-4)

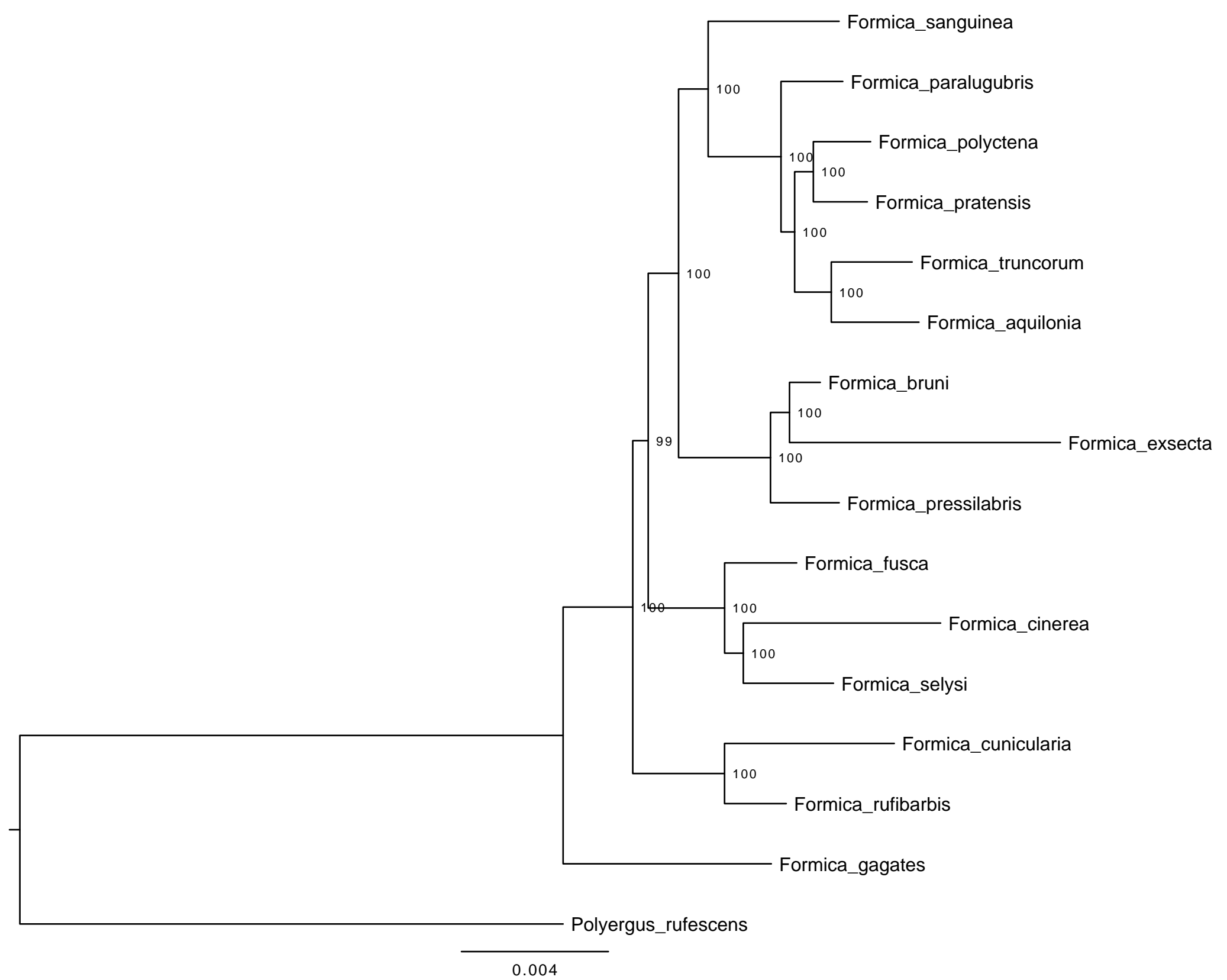

Supplement: Supplementary file 1 — Figure S1 Phylogenetic tree of the CLEAN supermatrix (970,619 bp) built using RAxML (GTR + GAMMA model, 500 bootstrap replications). (PDF 2 kb) [file 12862_2018_1159_MOESM1_ESM.pdf]

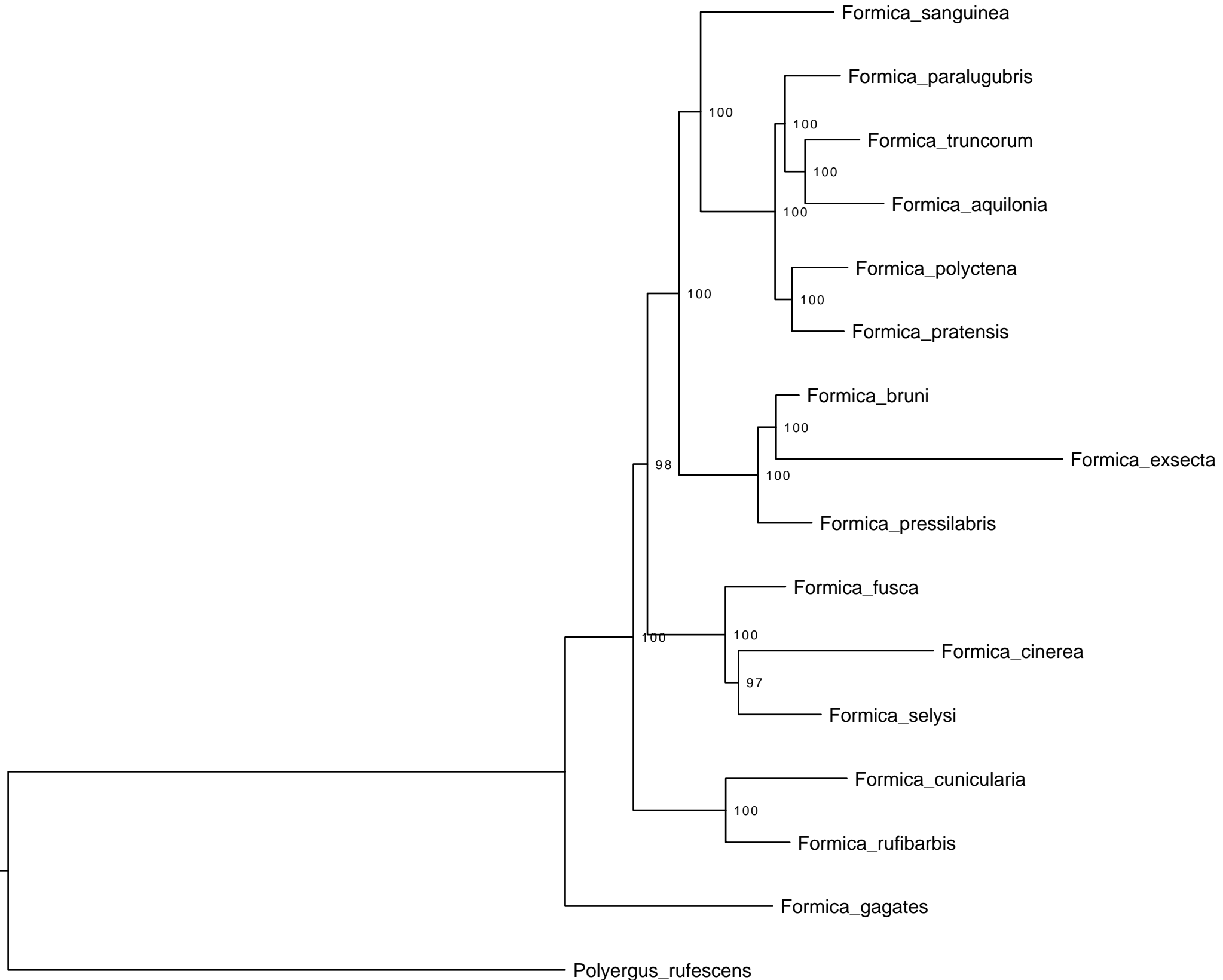

0.003

Supplement: Supplementary file 2 — Figure S2 Phylogenetic tree of the GAPLESS supermatrix (621,307 bp) built using RAxML (GTR + GAMMA model, 500 bootstrap replications). (PDF 2 kb) [file 12862_2018_1159_MOESM2_ESM.pdf]

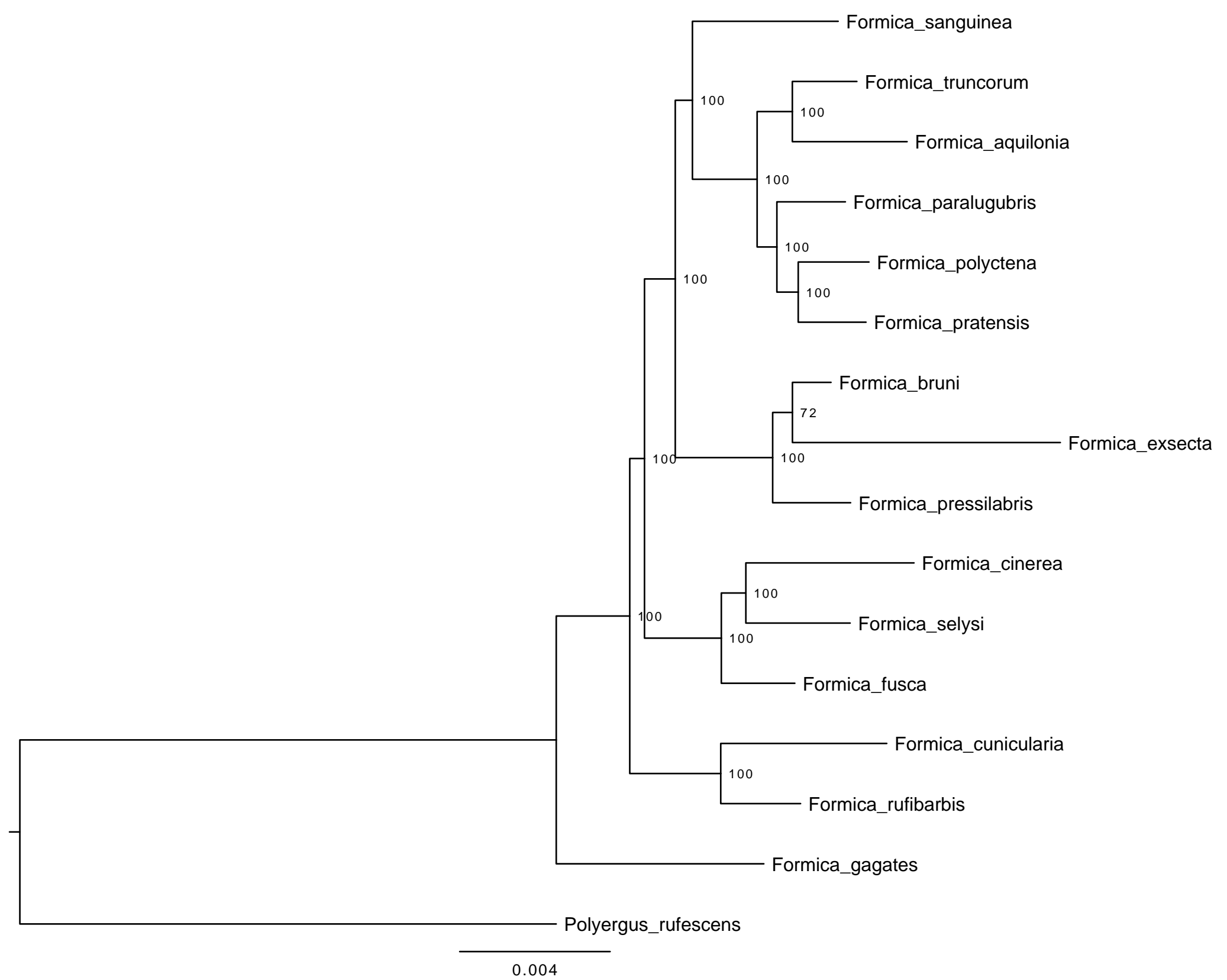

Supplement: Supplementary file 3 — Figure S3 Phylogenetic tree of the GCPOOR supermatrix (647,706 bp) built using RAxML (GTR + GAMMA model, 500 bootstrap replications). (PDF 2 kb) [file 12862_2018_1159_MOESM3_ESM.pdf]

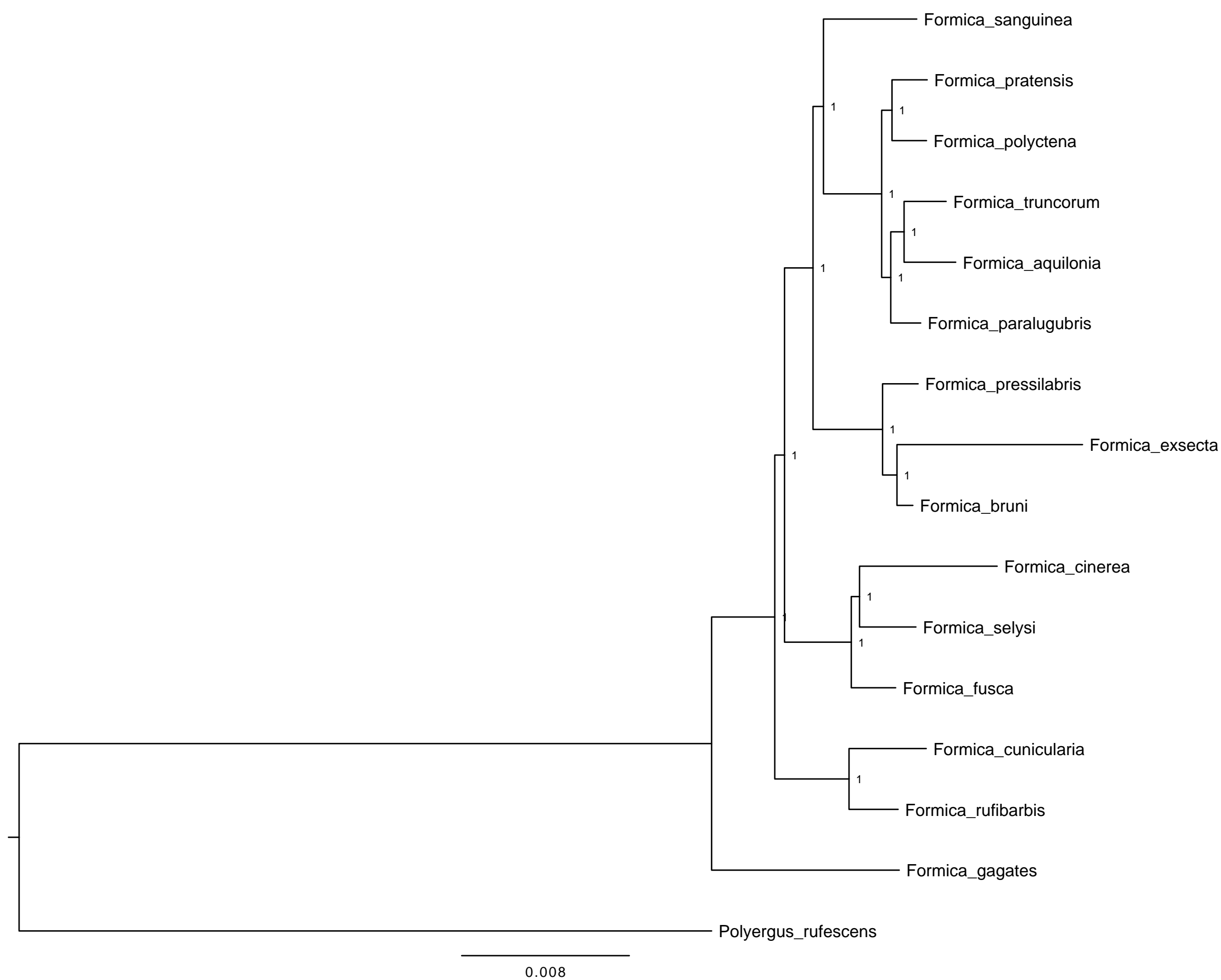

Supplement: Supplementary file 4 — Figure S4 Phylogenetic tree of the GAPLESS supermatrix (621,307 bp) built using PhyloBayes (two independent Markov chains, 15,000 generations). (PDF 2 kb) [file 12862_2018_1159_MOESM4_ESM.pdf]

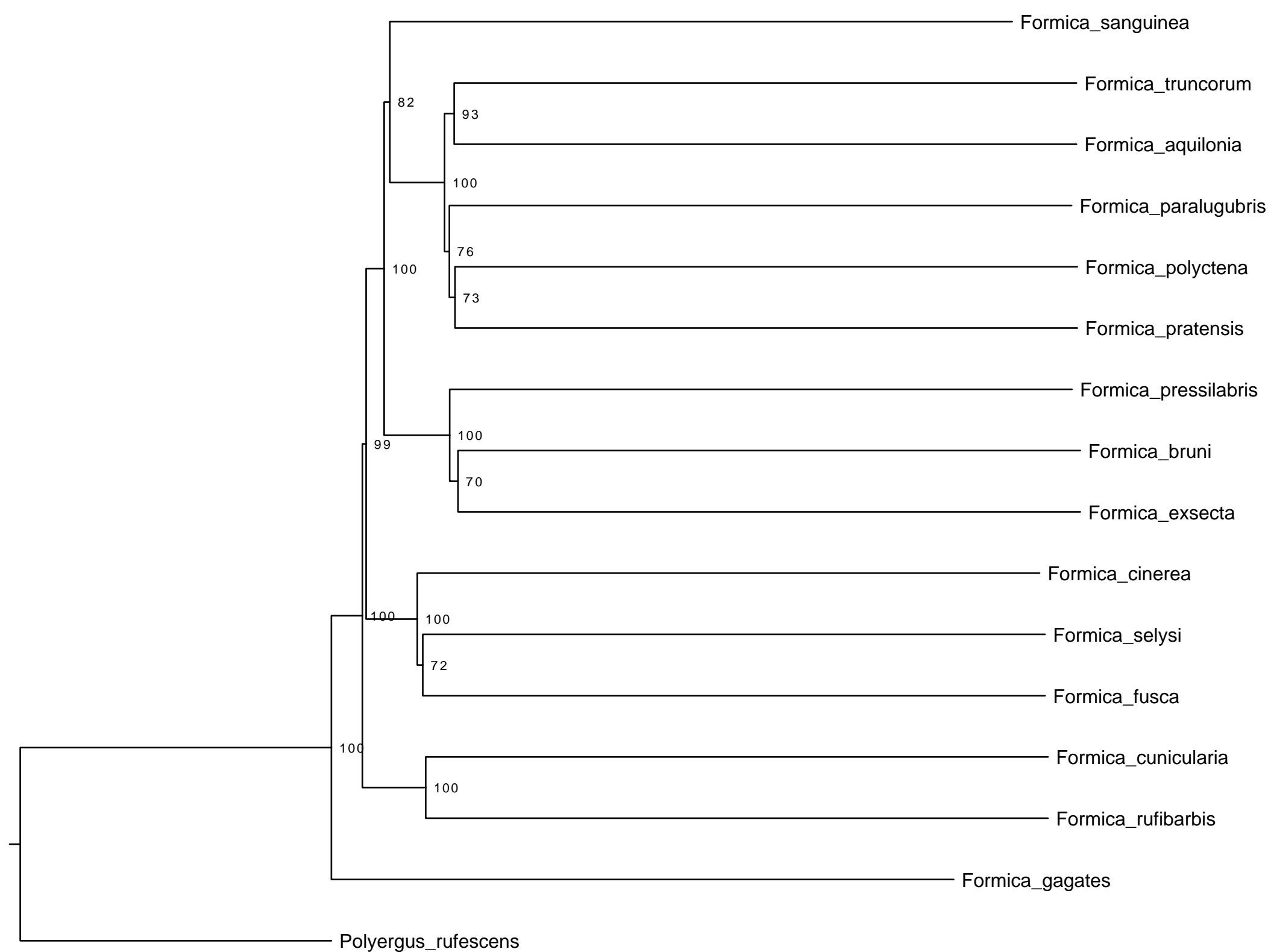

2.0

Supplement: Supplementary file 5 — Figure S5 Phylogenetic tree of the MP-EST analysis based on 945 gene trees (500 bootstrap replications for each gene tree). (PDF 2 kb) [file 12862_2018_1159_MOESM5_ESM.pdf]

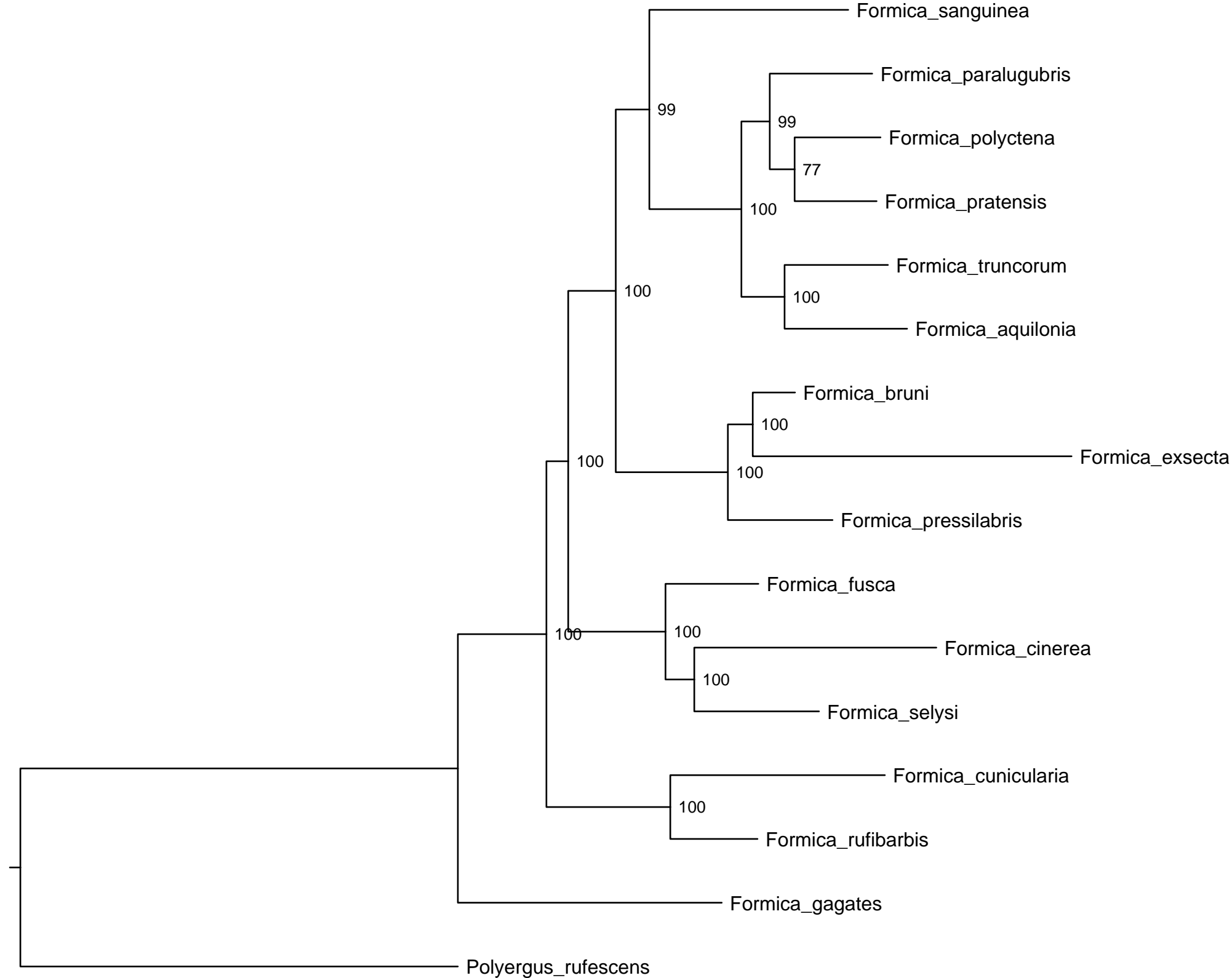

0.003

Supplement: Supplementary file 6 — Figure S6 Phylogenetic tree of the ALLPOSITIONS supermatrix (1270,080 bp) built using RAxML by partitioning the supermatrix by codon positions (GTR + GAMMA model, 500 bootstrap replications). (PDF 2 kb) [file 12862_2018_1159_MOESM6_ESM.pdf]

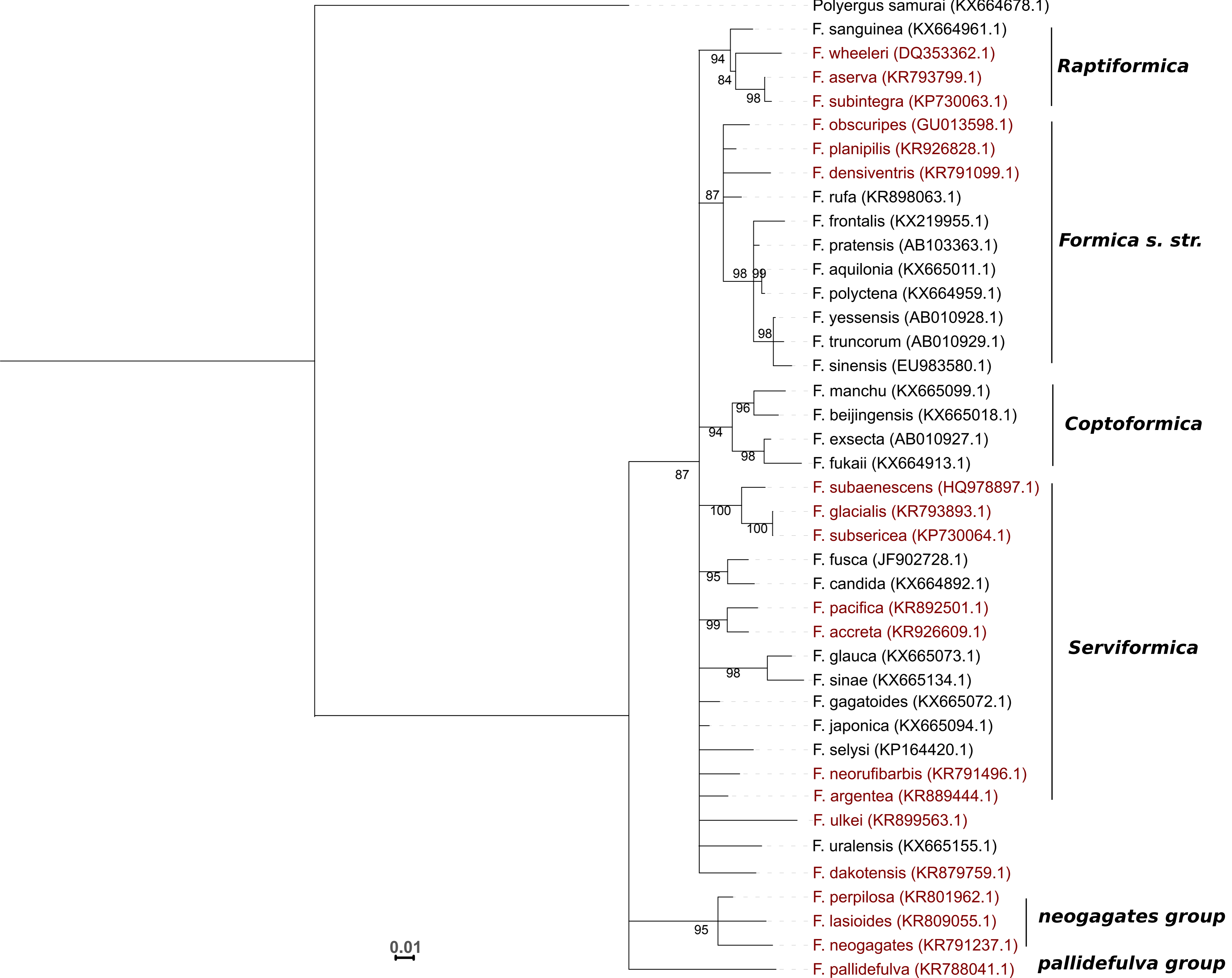

Supplement: Supplementary file 7 — Figure S7 Phylogenetic tree based on the cox1 mitochondrial gene of 41 Formica species borrowed from GeneBank (NCBI ID indicated between parentheses). The tree was built using RAxML (GTR + GAMMA, 500 bootstrap replications). Nodes supported by a bootstrap inferior to 70 were removed. Nearctic species are highlighted in red. (PDF 35 kb) [file 12862_2018_1159_MOESM7_ESM.pdf]
